# Lipid nanoparticle encapsulated TLR3 agonist adjuvant elicits potent T cell immunity against cancer and viruses

**DOI:** 10.1038/s41541-025-01349-w

**Published:** 2025-12-23

**Authors:** Kwang Hyun Ko, Seung-Hwan Lee, Young-Ho Choi, Soon Myung Kang, Hyun-Suk Yang, So Min Lee, Eun Bi Jo, Hyun Shik Bae, Seung-Beom Hong, Dong-Ho Kim, Seung Bin Cha

**Affiliations:** R&D Center, NA Vaccine Institute, Seoul, Republic of Korea

**Keywords:** Biotechnology, Immunology

## Abstract

Potent cellular immune responses are crucial for the development of effective vaccines against cancer and chronic infectious diseases. Here, we formulate Nexavant (NVT), a well-characterized TLR3 agonist, into lipid nanoparticles (LNPs) using either the ionizable lipid SM-102 or the cationic lipid DOTAP, and characterize their physicochemical properties and adjuvant potential. Both formulations achieve high encapsulation efficiency and enhance cellular uptake. In contrast to the stronger in vitro potency of DOTAP-based NVT/LNPs, SM-102–based NVT/LNPs (NVT/SM-LNPs) induce greater dendritic cell activation, cytokine production, and systemic T cell responses in vivo, likely due to more efficient delivery of NVT to the spleen. As an adjuvant for peptide vaccines, NVT/SM-LNP enhances antigen-specific CD4⁺ and CD8⁺ T cell responses and demonstrates potent therapeutic efficacy across subcutaneous, orthotopic, and metastatic TC-1 and B16-OVA tumor models, while also reducing viral titers in a chronic LCMV infection model. Compared to conventional adjuvants (poly(I:C), CpG, GM-CSF, IFA) and current mRNA vaccine platforms at clinically relevant doses, NVT/SM-LNP elicits stronger T cell immunity and enables effective neoantigen responses without requiring peptide-carrier conjugation. These findings establish NVT/SM-LNP as a potent adjuvant for T cell–targeted vaccines, with the lipid composition critically influencing immune targeting and efficacy, thereby guiding the design of next-generation vaccines.

## Introduction

T cells play a critical role in medicine, particularly in cancer, where they are essential for eliminating abnormal or malignant cells. However, the effective induction of T cell responses depends on the activation of innate immune mechanisms, which provide crucial signals for antigen recognition, costimulation, and the priming of adaptive immunity^1^. This interplay between innate and adaptive immunity forms the basis for designing immunotherapies that can effectively activate T cells, such as adjuvanted vaccines and immune-stimulatory platforms.

Nexavant (NVT) is a unique TLR3 and RIG-I agonist that does not activate MDA5^2,3^. When used as a vaccine adjuvant, NVT promotes robust Th1-type responses and cytotoxic T lymphocyte (CTL) activation, which are essential for antitumor immunity^2^. In previous studies, NVT has demonstrated anticancer efficacy by stimulating strong innate immune responses^3^, and it has also shown protective efficacy in mucosal vaccines, such as influenza, by inducing potent cellular immunity^4^. Despite its immunological potency, the in vivo application of NVT remains limited due to its poor delivery efficiency and susceptibility to rapid degradation by nucleases. Given that TLR3 and RIG-I, the target receptors of NVT, are located intracellularly, naked NVT has limited ability to reach these receptors. Consequently, there is a clear need for an optimized delivery system that can protect NVT from enzymatic degradation and promote its intracellular uptake and receptor engagement.

Lipid nanoparticles (LNPs) offer a powerful solution to this challenge. Originally developed for nucleic acid delivery, LNPs encapsulate RNA or DNA molecules, protect them from degradation, and facilitate their cellular uptake through endocytosis and endosomal escape^5,6^. LNP platforms have been successfully applied in mRNA vaccines and siRNA therapeutics and are increasingly explored in cancer immunotherapy, including non-viral gene delivery and CAR-T engineering^7^. In addition to their delivery capabilities, certain LNP formulations exhibit intrinsic immunostimulatory properties, acting as adjuvants themselves^8,9^. These dual functions—cargo delivery and immune activation—make LNPs highly attractive for next-generation vaccine development.

Given the intracellular localization of NVT’s target receptors and the dual functionality of LNPs, we hypothesized that encapsulating NVT in LNPs (NVT/LNPs) could synergistically enhance its delivery, stability, and immunogenicity. Specifically, LNPs could protect NVT from degradation and facilitate its cytosolic delivery, thereby maximizing its ability to activate innate immune signaling pathways. Furthermore, LNPs themselves may act as adjuvants to further amplify immune responses.

In this study, we systematically evaluated the immunostimulatory potential of NVT/LNPs formulated with various ionizable and cationic lipids. We assessed their ability to induce innate and adaptive immune responses, including peptide-specific CTL activation in vivo. Our findings demonstrate that optimized NVT/LNP formulations elicit stronger cellular immunity compared to conventional adjuvants, highlighting their potential as a next-generation platform for cancer vaccine development.

## Results

### In vitro characterization of LNP-encapsulated NVT (NVT/LNP) formulated with ionizable lipid SM-102 or cationic lipid DOTAP

**Fig. 1:**
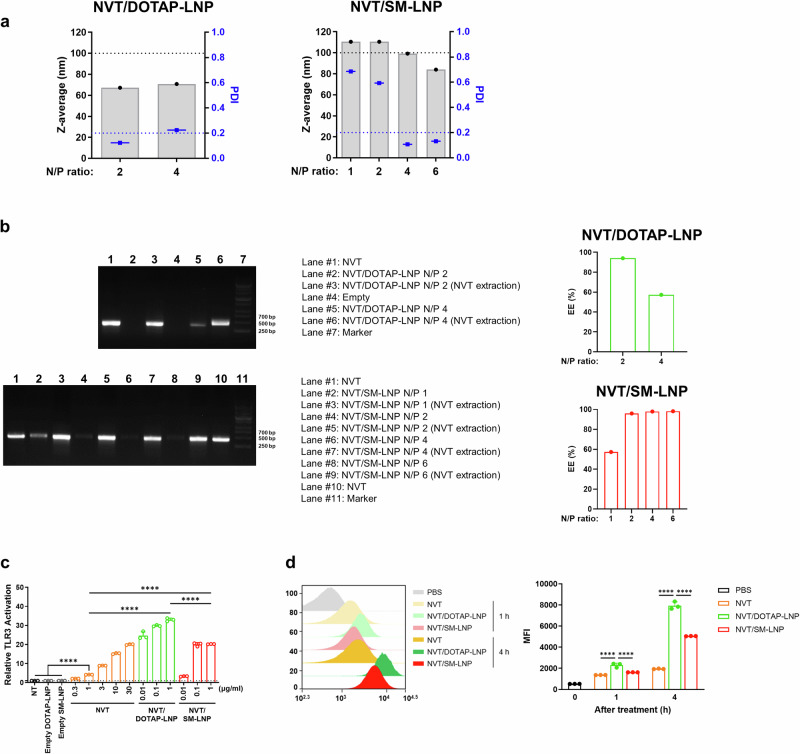
Characterization of NVT/LNP formulations. **a** Particle size and polydispersity index (PDI) of NVT/DOTAP-LNP and NVT/SM-LNP with different N/P ratios were characterized by dynamic light scattering. **b** Agarose gel electrophoresis and encapsulation efficiency (EE) analyses of NVT/DOTAP-LNP (upper) and NVT/SM-LNP (lower) at various N/P ratios. Free NVT, LNPs (before and after extraction), empty, and marker controls were loaded as indicated. NVT extraction was performed using ammonium acetate and isopropanol. EE values for each N/P ratio are shown in the right panel. Corresponding uncropped gel images are provided in Supplementary Fig. 1. Note that the main figure presents selected lanes for clarity. **c** TLR3 activity of NVT, NVT/DOTAP-LNP, NVT/SM-LNP, and empty LNP was in the HEK-Blue hTLR3 reporter cell line. The activation of NF-κB/AP-1 is shown as the fold change compared to the non-treated (NT) control. **d** RAW264.7 cells were incubated with IR750-labeled NVT, NVT/DOTAP-LNP, or NVT/SM-LNP for 1 or 4 h, and cellular uptake was evaluated by measuring the mean fluorescence intensity (MFI) of IR750. Representative plot data from three independent experiments are presented. **c**, **d** The data are presented as mean ± SD. Statistical analyses were performed using one-way ANOVA with Tukey’s multiple comparisons test. ****P < 0.0001.

NVT is a 424 bp dsRNA with well-studied properties and characteristics^2^. We prepared NVT/LNP using a microfluidizer with a molar ratio of 46.3:9.4:42.7:1.6 for ionizable lipid: DSPC: Cholesterol: DMG-PEG2000, matching the ratios used in the Pfizer-BioNTech mRNA vaccine^10,11^. For the ionizable lipid, we utilized SM-102, which is commonly used in mRNA vaccines. Additionally, we explored cationic lipids, such as DOTAP, which is also widely used as an LNP for mRNA delivery^12^, in the production of NVT/LNPs. Initially, we tested several N/P ratios to identify the most efficient encapsulation conditions. When SM-102 was used as the ionizable lipid (NVT/SM-LNP), the particle size was slightly less than 100 nm. The PDI dropped below 0.2, indicating homogeneity, at the N/P ratios above 4 (Fig. 1a). In the case of DOTAP (NVT/DOTAP-LNP), the size was slightly smaller, approximately 70 nm, and at an N/P ratio of 2, the PDI value fell below 0.2, confirming a more homogeneous manufacturing process compared to the N/P ratio of 4 (Fig. 1a). To evaluate the encapsulation efficiency of NVT into LNPs, we performed gel electrophoresis using naked NVT, NVT/LNP, and NVT extracted from LNPs. Naked NVT appeared as a distinct 424 bp band, while NVT/LNP showed no visible band due to the encapsulated NVT being retained in the gel well. After extracting NVT from the LNPs, the band reappeared, confirming successful encapsulation and recovery. Comparing band intensities before and after extraction allowed estimation of the encapsulation efficiency, which exceeded 90% at N/P ratios of 2 or 4 (Fig. 1b and Supplementary Fig. 1). Based on these results, we selected an N/P ratio of 4 for NVT/SM-LNP and 2 for NVT/DOTAP-LNP in subsequent experiments. The zeta potential values at N/P ratios of 4 for NVT/SM-LNP and 2 for NVT/DOTAP-LNP were measured as −3.8 mV and +1.2 mV, respectively. As expected, the formulation containing DOTAP, a cationic lipid, exhibited a positive surface charge. To assess whether NVT encapsulated with ionizable or cationic lipids exhibited superior activity, we compared their effects using a TLR3 reporter cell line. NVT/SM-LNP demonstrated approximately 300-fold potency compared to naked NVT. At the same time, NVT/DOTAP-LNP showed approximately 30,000-fold potency (Fig. 1c). Notably, NVT/DOTAP-LNP exhibited about 100 times stronger potency than NVT/SM-LNP. In the in vitro uptake assay, which involved capturing fluorescently labeled NVT with LNPs, we confirmed that a larger amount of NVT was taken up when enclosed in LNPs (Fig. 1d). Similar to the data from the reporter cell line, a stronger fluorescence signal was detected in the group treated with NVT/DOTAP-LNP compared to the group treated with NVT/SM-LNP, indicating greater uptake of NVT. In summary, we demonstrated that NVT was more effectively delivered into cells when encapsulated in LNPs, providing a logical basis for anticipating differences in efficacy in vivo.

### In vivo innate immune response induced by NVT/LNP

After confirming that NVT/LNP showed superior activity compared to naked NVT in vitro, we investigated whether it also improved the function of NVT in vivo. One of the primary functions of NVT is to activate dendritic cells (DCs) upon in vivo administration and to stimulate the innate immune system, including the secretion of type I interferon and IL-6^2^. For this reason, we utilized these as markers to judge the improved efficacy of LNP-encapsulated NVT. After intramuscular administration of each substance to mice, draining lymph nodes and spleens were isolated 24 hours later, and the degree of DC activity was evaluated.

**Fig. 2:**
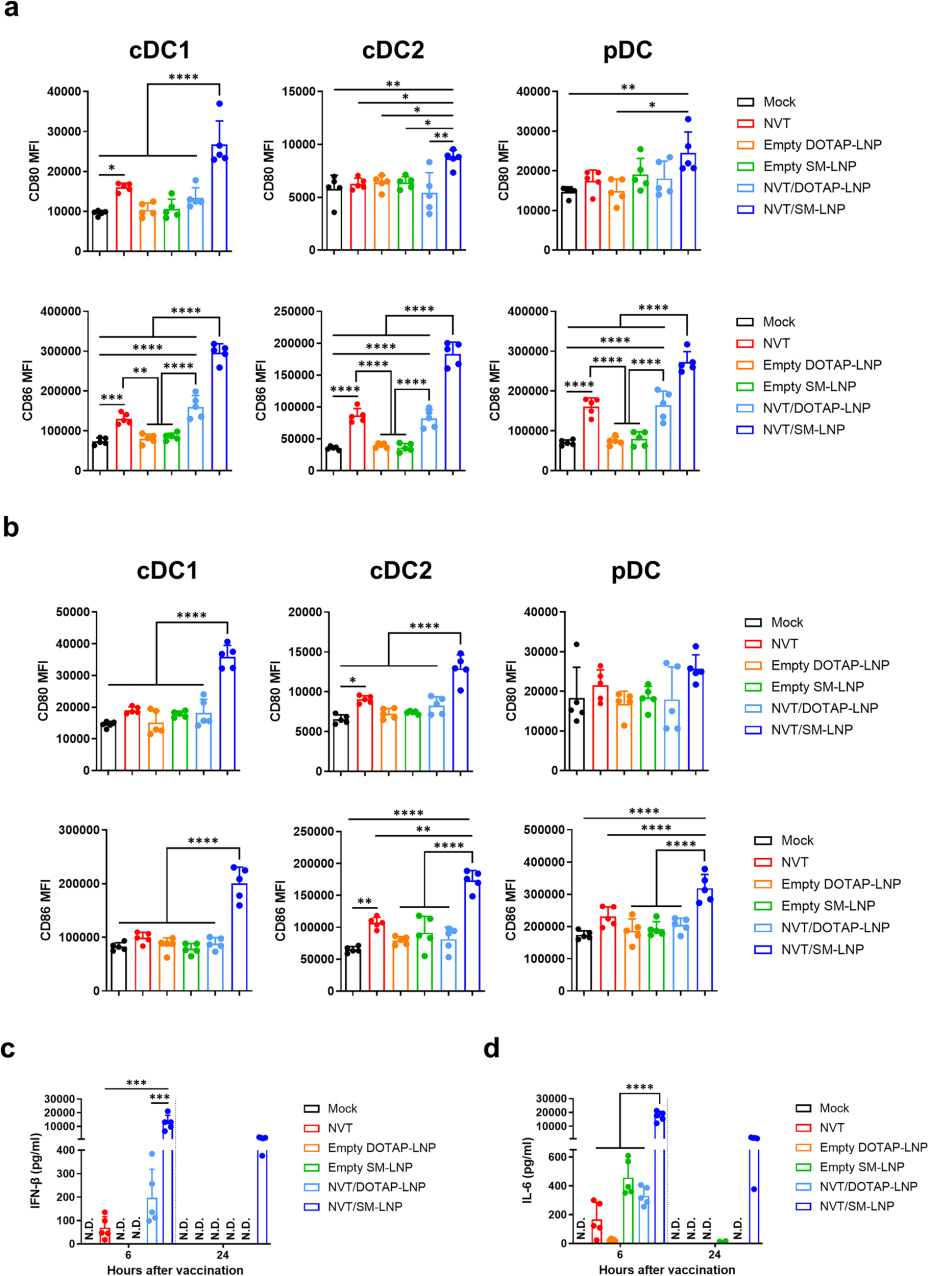
Innate immune response of NVT/LNP. C57BL/6 mice were immunized intramuscularly with 10 μg of NVT, NVT/DOTAP-LNP, NVT/SM-LNP, or equivalent amounts of empty DOTAP-LNP or empty SM-LNP. **a**, **b** The MFI of CD80 and CD86 in the cDC1 (CD11c^+^MHC-II^+^XCR1^+^CD8^+^), cDC2 (CD11c^+^MHC-II^+^XCR1^−^), and pDC (CD11c^+^MHC-II^+^B220^+^) subpopulations in (**a**) inguinal lymph nodes and (**b**) spleen at 24 h post-immunization was measured by flow cytometry. *n* = 5. **c**, **d** Serum levels of (**c**) IFN-β and (**d**) IL-6 at 6 and 24 h post-immunization were measured using ELISA. *n* = 5. The data are presented as mean ± SD. Statistical analyses were performed using one-way ANOVA with Tukey’s multiple comparisons test. N.D. not detected; *P < 0.05; **P < 0.01; ***P < 0.001; ****P < 0.0001.

Consistent with previous findings^2^, naked NVT activated DCs across all subsets in the draining lymph nodes. Both NVT/DOTAP-LNP and NVT/SM-LNP activated DCs; however, the activation induced by NVT/DOTAP-LNP was comparable to that of naked NVT, while NVT/SM-LNP demonstrated significantly stronger DC activation than either naked NVT or NVT/DOTAP-LNP. Additionally, empty LNP showed no DC activation function. In the spleen, DC activation was relatively weak in the groups administered naked NVT or NVT/DOTAP-LNP. In contrast, strong DC activation was observed only in the group administered NVT/SM-LNP (Fig. 2a, b and Supplementary Fig. 2). As in the draining lymph node, empty LNP hardly activated DCs. To confirm that the robust DC activation by NVT/SM-LNP was dependent on NVT, we tested the level of DC activation at various doses. It was confirmed that the activation of DCs by NVT/SM-LNP occurred in a dose-dependent manner (Supplementary Fig. 3). The observed upregulation of costimulatory molecules CD80 and CD86 indicates that NVT/SM-LNP promotes a more immunogenic phenotype in DCs. These molecules provide essential secondary signals for T cell priming, and their increased expression is closely linked to enhanced antigen-presenting capacity and the subsequent initiation of adaptive immune responses. To assess whether these observations could be generalized to other ionizable lipids, we evaluated the DC activation ability of NVT formulated with several commercially available candidates, including DODMA, DODAP, DORI, DLIN-KC2, and DLIN-MC3. Consistent with our findings for DOTAP and SM-102, DODMA, DODAP, and DORI displayed profiles similar to DOTAP, while DLIN-KC2 and DLIN-MC3 exhibited characteristics more closely aligned with SM-102 (Supplementary Figs. 4, 5).

Measurement of IFN-β and IL-6 levels revealed significantly higher cytokine levels in the NVT/SM-LNP group at 6 hours post-administration. However, 24 h after administration, it returned to almost basal levels (Fig. 2c, d). In the case of IFN-β, trace amounts were detected only in groups containing NVT. It was not induced in the group administered empty LNP or NVT/DOTAP-LNP (Fig. 2c). We confirmed that IL-6 was induced in small amounts not only in the group containing NVT but also in the group administered empty LNP (Fig. 2d). This is consistent with the previous study results that LNP itself has some immunogenicity^9^. Empty LNP alone did not induce significant changes in innate immunity, except for a minor increase in IL-6 secretion. In contrast, the response triggered by naked NVT was amplified, indicating that LNP encapsulation enhances potency by preserving NVT’s existing immune induction pathway and improving its intracellular delivery. Interestingly, NVT/DOTAP-LNP showed the most robust activity in vitro, but did not show significant improvement in vivo compared to naked NVT. In summary, NVT encapsulated in SM-LNP exhibited potent innate immune activity, whereas when encapsulated in DOTAP-LNP, it did not. The innate immune activation observed in vivo did not correlate with the level of activity measured in the reporter cell line in vitro.

### Cell-mediated immune response with a peptide vaccine adjuvanted with NVT/LNP

**Fig. 3:**
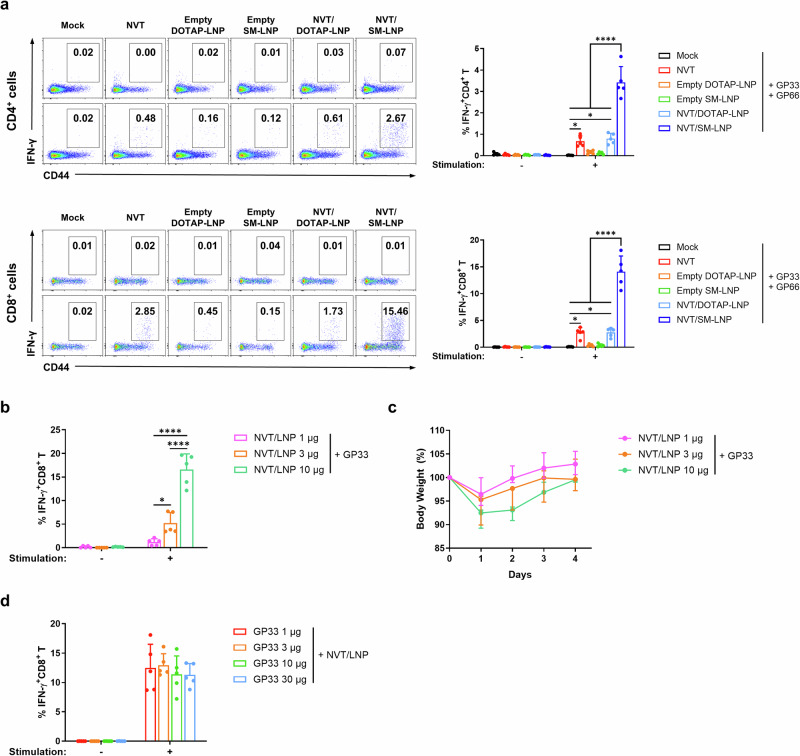
Adaptive immune response of NVT/LNP. **a** C57BL/6 mice were immunized intramuscularly with 10 μg of GP33 and 10 μg of GP66 formulated with 10 μg of NVT, NVT/DOTAP-LNP, NVT/SM-LNP, or equivalent amounts of empty DOTAP-LNP or empty SM-LNP on days 7, 14, and 21. On day 28, GP33-specific CD8^+^ T cell responses and GP66-specific CD4^+^ T cell responses in PBMCs were measured by flow cytometry. *n* = 5. **b** C57BL/6 mice were immunized intramuscularly with 10 µg of GP33 formulated with 1, 3, or 10 µg of NVT/LNP, and GP33-specific CD8^+^ T cell responses in PBMCs were measured by flow cytometry. *n* = 5. **c** Body weight of C57BL/6 mice was monitored from day 0 to day 4 following the first intramuscular immunization with GP33 formulated with 1, 3, or 10 µg of NVT/LNP as described in (**b**). Body weight is expressed as a percentage of the baseline weight on day 0. Data are presented as median (min–max). *n* = 5. **d** C57BL/6 mice were immunized intramuscularly with 1, 3, 10, or 30 µg of GP33 formulated with 10 µg of NVT/LNP, and GP33-specific CD8^+^ T cell responses in PBMCs were measured by flow cytometry. *n* = 5. The data are presented as mean ± SD. Statistical analyses were performed using one-way ANOVA with Tukey’s multiple comparisons test. *P < 0.05; ****P < 0.0001.

It is widely recognized that DC activation is essential for inducing cellular immune responses^13^. To investigate whether the enhanced innate immune activation observed with LNP-encapsulated NVT leads to improved adaptive immune responses, we conducted a series of experiments. We utilized GP66 (DIYKGVYQFKSV) and GP33 (KAVYNFATC), which are well-known CD4 and CD8 epitopes of mouse LCMV, as peptide antigens^14^. To compare antigen-specific cellular immunity among adjuvants, we immunized mice intramuscularly three times at one-week intervals with GP66 and GP33 peptide antigen with NVT, empty LNP, or NVT/LNP. One week after the last vaccination, mouse PBMCs were stimulated with the administered peptides to measure antigen-specific T cell responses. Consistent with the DC activation results, the group receiving NVT/SM-LNP as an adjuvant demonstrated the highest CD4^+^ and CD8^+^ T cell responses, while empty LNP induced minimal cellular immune responses. Notably, CD4^+^ and CD8^+^ T cell responses were also observed with NVT/DOTAP-LNP, but there was no significant improvement compared to naked NVT (Fig. 3a). When T cell responses were measured using other ionized lipids, the results confirmed those of the previously observed innate immune response (Supplementary Fig. 6). Given these results, we chose NVT/SM-LNP as the adjuvant for the peptide vaccine in subsequent experiments to assess the vaccine’s efficacy, and named it NVT/LNP.

Next, we performed a dose optimization experiment. In this experiment, mice were vaccinated with 10 µg of GP33 and 1, 3, or 10 µg of NVT/LNP for NVT/LNP dose optimization, and with 1, 3, 10, or 30 µg of GP33 and 10 µg of NVT/LNP for peptide dose optimization. The peptide epitope-specific cellular immune response showed a dose-dependent increase with NVT/LNP (Fig. 3b). As part of the NVT/LNP dose optimization, body weight was monitored from day 0 to day 4 following the first immunization to assess potential toxicity. A transient, dose-dependent weight loss was observed on day 1 post-vaccination, followed by recovery to baseline weight within two to three days (Fig. 3c). Although slightly higher T cell responses were observed at 1 and 3 µg peptide doses compared to 10 and 30 µg doses, these differences were not statistically significant, and all doses elicited comparable antigen-specific activation (Fig. 3d). Therefore, we selected 10 µg of peptide to ensure sufficient antigen availability, reproducibility by minimizing variability, and consistency with widely used dosing in murine peptide vaccination models^15,16^. Accordingly, 10 µg of peptide and 10 µg of LNP were used as the optimal doses throughout the study, unless otherwise stated. In conclusion, we demonstrated that the increased innate immunity induced by NVT/LNP resulted in enhanced cellular immune responses, with variations in these responses depending on the components of the ionized lipids comprising the LNPs.

### Therapeutic efficacy of peptide vaccines adjuvanted with NVT/LNP for cancer and chronic viral infection

**Fig. 4:**
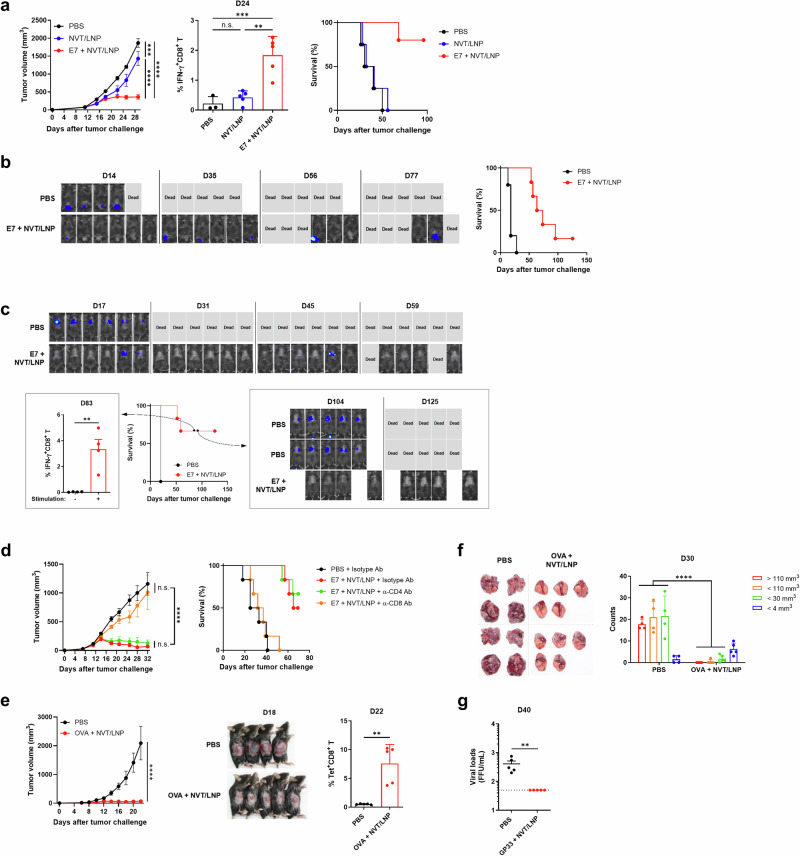
Therapeutic effects of NVT/LNP-based peptide vaccines against cancer and chronic viral infection. Mice were intramuscularly vaccinated with 10 μg of NVT/LNP-formulated peptide vaccines (10 μg of peptide antigen) on days 7, 14, and 21 in tumor models. Tumor progression and survival were monitored over time. **a** C57BL/6 mice were subcutaneously injected with 5 × 10^5^ TC-1-Luc cells and treated with NVT/LNP containing or lacking the E7 peptide. On day 24, E7-specific CD8^+^ T cell responses were assessed from isolated PBMCs. *n* = 4, 5, 6. This experiment was independently repeated three times, and data from one representative experiment are shown. **b** In the cervical cancer model, mice were intravaginally challenged with 5 × 10^5^ TC-1-Luc cells and immunized with E7 peptide-formulated NVT/LNP, *n* = 6, or PBS control, *n* = 5. Representative bioluminescence images of mice were captured using a VISQUE® Smart-LF imaging system. **c** For the lung metastasis model, mice were intravenously injected with 5 × 10^5^ TC-1-Luc cells and vaccinated as in (**b**). E7-specific CD8^+^ T cell responses were analyzed from surviving mice on day 83, followed by tumor rechallenge on day 90 via tail vein injection. *n* = 6 (1^st^ challenge); 10, 4 (2^nd^ challenge). Representative bioluminescence images of mice were captured using a VISQUE® Smart-LF imaging system. This experiment was independently repeated twice, and data from one representative experiment are shown. **d** Tumor-bearing mice were treated with NVT/LNP vaccines intramuscularly, and anti-CD4 or anti-CD8 antibodies (200 µg) were administered intraperitoneally twice weekly from day 7, for a total of six doses. *n* = 6. **e** C57BL/6 mice were subcutaneously inoculated with 5 × 10^5^ B16F10-OVA cells and vaccinated with OVA CD8 peptide-formulated NVT/LNP. Tumor burden at day 18 is indicated by a red dotted line, and OVA-specific CD8^+^ T cells were quantified on day 22 by tetramer staining of PBMCs. Representative images of mice were captured using a digital camera prior to tumor measuring on day 18. *n* = 5. This experiment was independently repeated three times, and data from one representative experiment are shown. **f** Following intravenous injection of 5 × 10^5^ B16F10-OVA cells, mice were vaccinated with OVA peptide-formulated NVT/LNP. Lung tumor burden was determined on day 30 by counting tumor nodules after sacrifice. Representative images of lung were captured using a digital camera prior to nodule counting. *n* = 4, 5. This experiment was independently repeated twice, and data from one representative experiment are shown. **g** C57BL/6 mice were infected with LCMV and immunized intramuscularly with GP33 peptide-formulated NVT/LNP at 14-, 21-, and 28-days post-infection. Viral titers in the blood were measured on day 40. *n* = 5. Data are shown as mean ± SEM for tumor growth, and mean ± SD for T cell responses and viral titers. Statistical significance was determined using one-way ANOVA with Tukey’s multiple comparisons test. **P < 0.01; ***P < 0.001; ****P < 0.0001; n.s. not significant.

To determine whether the potent cellular immune response induced by NVT/LNP results in functional improvements, we evaluated its therapeutic efficacy in various anticancer and LCMV virus infection models. We utilized the mouse syngeneic TC-1 model, which expresses the E7 protein of HPV16 (Human papillomavirus), to assess the efficacy of the cancer therapeutic vaccine. The peptide E7 (RAHYNIVTF) served as the vaccine antigen. In experiments confirming the therapeutic effect across all cancer models shown in Fig. 4, the vaccine was administered three times on Day 7, 14, and 21, relative to the day the cancer was implanted in the mouse (Day 0). Imaging, size measurements, and immunoassays were performed on the indicated days.

The anticancer efficacy in the TC-1 model was verified in subcutaneous, orthotopic, and metastatic models. In the subcutaneous model, evaluation of the peptide therapeutic vaccine demonstrated a dramatic reduction in tumor size compared to the control group (Fig. 4a). In the vaccinated group, tumor size decreased after the second vaccine dose, coinciding with the production of effector T cells. When we measured the E7-specific CD8^+^ T cell response in the PBMCs of the mice on Day 24, we detected the response only in the vaccinated group. All control mice died by Day 50, while 60% of the mice in the therapeutic vaccine group completely responded and survived for up to 100 days (Fig. 4a). Since NVT has anticancer effects^3^, we included a group that received only the adjuvant NVT/LNP, exhibiting some anticancer efficacy as expected. Because T cells were not induced and the efficacy was significantly lower than the vaccine group, the adjuvant-only group was excluded from subsequent experiments.

The subcutaneous model is straightforward but has several limitations compared to more realistic cancer models^17^. Therefore, we evaluated the efficacy of this peptide vaccine in both an orthotopic model and a metastatic model. In the orthotopic model, implanting TC-1 cells directly into the cervix, allowing for the study of tumor growth and response to therapies in a more relevant anatomical location than subcutaneous models. The median survival time in the vaccine group increased by approximately 3.8-fold compared to the PBS control group (68 days vs. 18 days), with one mouse (16%) achieving a complete response (Fig. 4b). In the metastatic model, the indicated number of cancer cells was injected intravenously to induce metastasis to the lung. All mice in the control group died within 20 days, while 66.7% of the vaccinated group showed a complete response (Fig. 4c). Antigen-specific CD8^+^ T cell responses were detected in the PBMCs of surviving mice even on day 83, and all of these individuals survived when re-challenged with tumor cells (Fig. 4c).

To confirm that the anticancer effects of the peptide vaccine were due to the vaccine-induced CD8^+^ T cell response, a T cell depletion experiment was conducted using anti-CD4 or anti-CD8 antibodies. Administration of depletion antibodies was initiated during the first vaccination and continued twice per week thereafter. We confirmed efficient depletion of CD4⁺ or CD8⁺ T cells in the respective treatment groups by flow cytometric analysis of PBMCs collected on day 18 (Supplementary Fig. 7). The anticancer effect persisted in the group administered isotype antibodies and in the group from which CD4^+^ T cells were depleted. The anticancer effect disappeared only in the experimental group where CD8^+^ T cells were depleted (Fig. 4d). This demonstrates that the anticancer effect was mediated by the antigen-specific CD8^+^ T cell response induced by the peptide vaccine.

To verify whether this anticancer effect is universally observed in other cancer types, we used the well-known CD8 peptide epitope (SIINFEKL) from the OVA protein in the B16-OVA cancer models, testing it in both subcutaneous and metastatic models. In the subcutaneous model, we confirmed that tumor size was significantly reduced, along with the peptide epitope-specific CD8^+^ T cell response (Fig. 4e). In the metastatic model, we compared the number of nodules that had metastasized to the lungs at autopsy on day 30 and found that both the number and size of nodules were significantly reduced in the group administered the vaccine (Fig. 4f).

Finally, we evaluated the therapeutic effect of the CD8 + T cell response induced by the peptide vaccine on chronic virus infection using the LCMV chronic virus model. GP33, a well-known CD8 epitope of the LCMV virus, was used as the vaccine antigen. After infection with LCMV CL13 to induce chronic viremia on day 0, the vaccine was administered on days 14 and 21. At day 40, approximately three weeks after administration, we measured the immune response in PBMC and the viral titer in the blood. Similar to the results from the anticancer treatment, the viral titer in the blood was significantly reduced (Fig. 4g). In summary, these experiments confirmed that when NVT/LNP, which induces a strong cellular immune response, was used as an adjuvant, it exhibited excellent therapeutic effects in both cancer and chronic virus therapy.

### Immunogenicity comparison with other adjuvants and mRNA vaccines

**Fig. 5:**
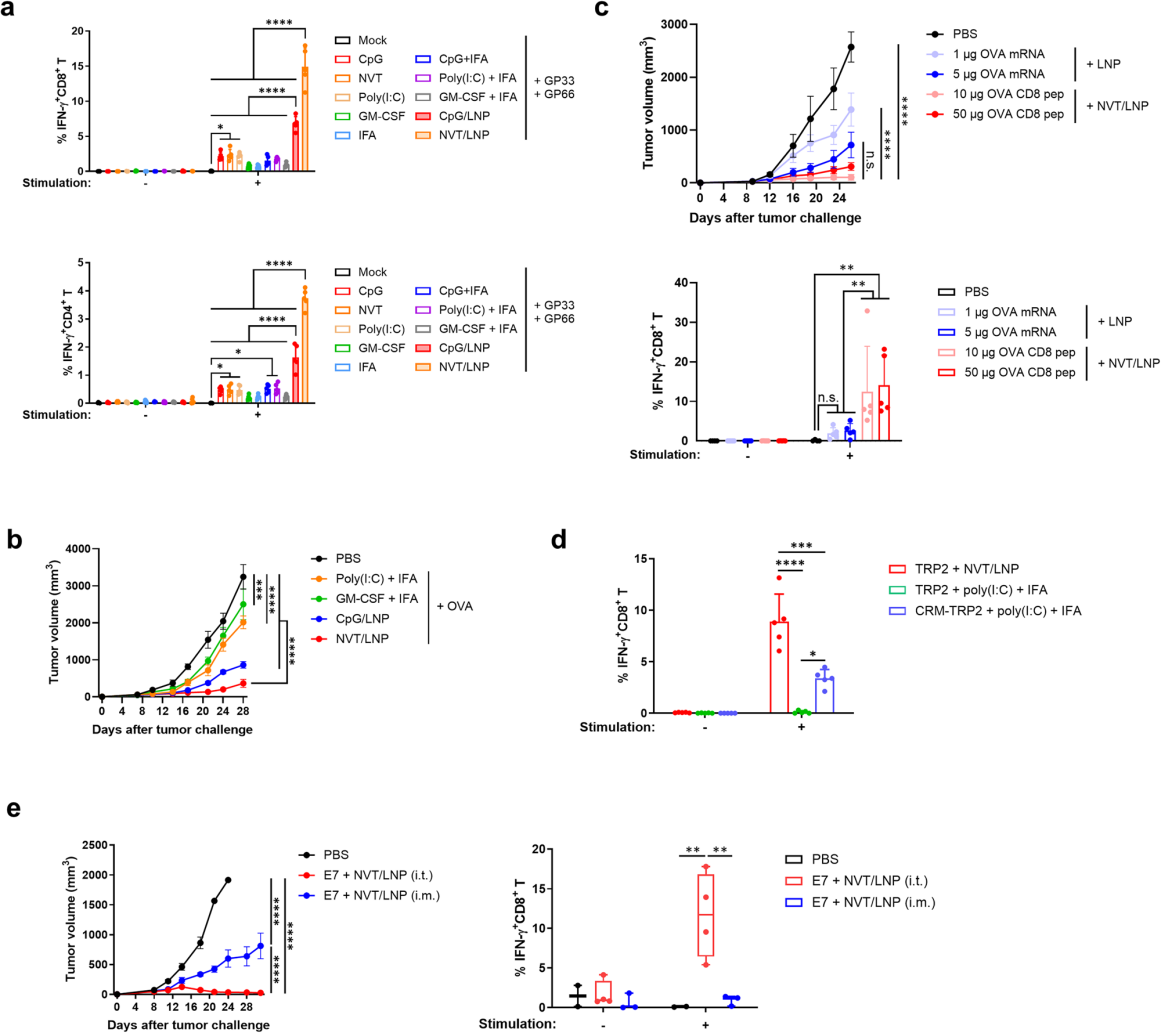
Comparison with adjuvants and methods used in the other peptide vaccine. **a** C57BL/6 mice were immunized intramuscularly on days 7, 14, and 21 with 10 μg each of GP33 and GP66 antigens, formulated with 10 μg of CpG, NVT, poly(I:C), GM-CSF, or with 10 μg of CpG, poly(I:C), or GM-CSF emulsified in IFA at a 1:1 (v/v) ratio with the antigens. Additional groups were administered 10 μg each of GP33 and GP66, formulated with 10 μg of CpG/LNP or NVT/LNP. On day 28, GP33-specific CD8^+^ T cell responses and GP66-specific CD4^+^ T cell responses in PBMCs were measured by flow cytometry. *n* = 5. **b** C57BL/6 mice were subcutaneously inoculated with 5 × 10^5^ B16F10-OVA cells, and each adjuvant was administered intramuscularly in combination with the OVA CD8 peptide on days 7, 14, and 21 post-tumor implantations, vaccinated as in (**a**). Tumor growth was monitored over time. *n* = 5. **c** C57BL/6 mice were subcutaneously inoculated with 5 × 10^5^ B16F10-OVA cells and vaccinated intramuscularly with OVA mRNA/SM-LNP or OVA peptide/NVT/SM-LNP vaccines on days 9, 16, and 23. For OVA mRNA antigen, doses of 1 or 5 µg were used, and for OVA peptide antigen, doses of 10 or 50 µg were applied. Tumor growth was monitored over time. On day 26, OVA-specific CD8^+^ T cell responses were measured from PBMCs. *n* = 5. **d** C57BL/6 mice were immunized intramuscularly with 10 μg each of TRP2 antigens, formulated with 10 μg of NVT/LNP or 10 μg of poly(I:C) emulsified in IFA at a 1:1 (v/v) ratio with the antigens on days 7, 14, and 21. Additional groups were administered 10 μg of CRM197-conjugated TRP2 (CRM-TRP2), formulated with 10 μg of poly(I:C) emulsified in IFA at a 1:1 (v/v) ratio with the antigens. On day 28, TRP2-specific CD8^+^ T cell responses in isolated PBMCs were measured by flow cytometry. *n* = 5. **e** C57BL/6 mice were subcutaneously inoculated with 5 × 10⁵ TC-1-Luc cells on day 0. Mice were then vaccinated with E7 peptide-NVT/LNP vaccines (1 µg peptide + 1 µg NVT/LNP) either intramuscularly or intratumorally on days 7, 14, and 21 post-tumor inoculation. Tumor growth was monitored over time. On day 21, peripheral blood mononuclear cells (PBMCs) were collected to measure E7-specific CD8⁺ T cell responses. *n* = 4. Data are shown as mean ± SEM for tumor growth, and mean ± SD for T cell responses and viral titers. Statistical significance was determined using one-way ANOVA with Tukey’s multiple comparisons test. *P < 0.05; **P < 0.01; ***P < 0.001; ****P < 0.0001; n.s., not significant.

Next, we examined the differences in immunogenicity when NVT/LNP was used as an adjuvant for a peptide vaccine, compared to those used in existing clinical trials. To investigate this, we compared the immunogenicity of peptide vaccines that utilized poly(I:C), CpG, GM-CSF, and IFA—the most widely used adjuvants in current peptide vaccines—either individually or in combination, against peptide vaccines using NVT/LNP as the adjuvant. GP33 and GP66 served as peptide antigens, and the doses of each adjuvant were administered according to those referenced in existing studies^18–21^. Among the adjuvants previously used in peptide vaccines, CpG and poly(I:C) elicited significant antigen-specific CD4^+^ and CD8^+^ T cell responses, similar to those elicited by NVT. Interestingly, no significant antigen-specific cellular immune response was observed in the GM-CSF and IFA alone groups. Additionally, no synergistic effect on the immune response was observed when IFA was combined with other adjuvants. The CpG/LNP groups induced significantly higher T cell responses than the other adjuvant and IFA combination groups. However, these responses were still significantly lower than those observed in the NVT/LNP group (Fig. 5a). Taken together, NVT/LNP adjuvant induces potent antigen-specific CD4^+^ and CD8^+^ T cell responses.

To determine whether the differences in cellular immune responses between adjuvants translate to anticancer effects, we compared the anticancer effects in the B16-OVA model. When GM-CSF + IFA and poly(I:C) + IFA were used as adjuvants for peptide vaccines, a significant anticancer effect was observed compared to the control. In contrast, NVT/LNP induced a markedly more substantial anticancer effect, which appeared to correlate with the magnitude of the overall T cell response. CpG/LNP also elicited a more potent anticancer effect than the conventional adjuvants, but its efficacy remained lower than that of NVT/LNP (Fig. 5b).

We compared the efficacy of the mRNA vaccine platform, which has recently gained attention as a cancer treatment, with that of the vaccine platform using the NVT/LNP adjuvant. Due to differing modalities and dose-response correlations, accurate comparisons are challenging. To address this, we assessed efficacy at 0.02 human dose (HD) and 0.1 HD, referencing the HD used in commercially available mRNA vaccines and peptide vaccines in clinical trials^22–26^. For mRNA, 1 µg was used for the 0.02 HD dose and 5 µg for the 0.1 HD dose. For the peptide, 10 µg was used for the 0.02 HD dose and 50 µg for the 0.1 HD dose. Each vaccine was administered three times at one-week intervals, starting on day 7 (D7) after tumor implantation (D0). In the B16-OVA model, the mRNA vaccine demonstrated a dose-dependent anticancer effect. On day 20, when PBMCs from the corresponding individuals were isolated and analyzed, an OVA-specific CD8^+^ T cell response was confirmed. This finding aligns with previous research indicating that the mRNA vaccine platform induces CD8^+^ T responses and exhibits anticancer effects. The peptide vaccine showed a significantly reduced tumor size, accompanied by a potent antigen-specific CD8^+^ T cell response, compared to the mRNA vaccine groups (Fig. 5c). In both groups that received the peptide vaccine, a more substantial anticancer effect was observed compared to the 0.02 HD mRNA vaccine. However, no significant difference was found compared to the 0.1 HD mRNA vaccine. The T cell response measured is specific to the OVA CD8 epitope; therefore, it cannot be concluded that the overall immune response of the mRNA vaccine is lower. Considering the induction of a cellular immune response targeted to the used epitope, the peptide vaccine demonstrated superior efficacy compared to the mRNA platform.

Finally, we conducted comparative experiments using platforms where peptides were conjugated to carrier proteins. Peptides are considered safe because they contain only the minimal epitope region required to elicit an immune response. However, their primary drawback is low immunogenicity. To address this issue, numerous studies are underway to enhance their immunogenicity by conjugating them to carrier proteins such as CRM197^27,28^. Research has demonstrated significant improvements in immunogenicity, leading to the initiation of clinical trials. We hypothesized that using NVT/LNP as an adjuvant could induce sufficient immunity without the need for a complex conjugation process, and we conducted experiments to verify this. In this study, TRP2, a self-antigen with low immunogenicity^29^, was conjugated to CRM197 and administered to mice for comparison of immunogenicity. In the experimental group that received the conjugated vaccine, the dose was determined by 10 µg of TRP2 peptide conjugated to CRM197. When TRP2 was used as an adjuvant with poly(I:C) and IFA, both of which are widely used in peptide vaccines, no significant immune response was induced. In contrast, reactivity improved when TRP2 was conjugated with CRM197. When NVT/LNP was used as an adjuvant for the free epitope, the strongest immune response was induced, which is far exceeding the conjugated case (Fig. 5d). While further testing of the carrier protein’s additional advantages is necessary, the peptide-specific CTL response was significantly stronger without any complicated conjugation to the carrier protein.

Intratumoral administration of cancer vaccines is believed to offer an advantage by allowing direct antigen recognition by tumor-resident antigen-presenting cells within the tumor microenvironment, potentially leading to enhanced immunogenicity compared to other delivery routes^30^. Consistent with this, intratumoral injection of the NVT/LNP-based peptide vaccine resulted in pronounced tumor growth inhibition and robust CD8⁺ T cell responses. Remarkably, these effects approached complete tumor regression even in the absence of PD-1 blockade or other combination therapies (Fig. 5e). No detectable T cell response was observed in the intramuscular group in Fig. 5e, in contrast to the strong responses seen in Fig. 4c. This discrepancy likely stems from the lower dose of NVT/LNP used in Fig. 5e, which was intentionally reduced to better visualize route-dependent differences in vaccine efficacy. The decreased dose appears to have been insufficient to elicit a measurable immune response via intramuscular injection. Taken together, based on multiple examples with diverse epitopes, the NVT/LNP is a powerful T-cell adjuvant system that can be simply mixed with target peptides without any complicated chemical modification steps.

### Immunogenicity of peptide-based neoantigen adjuvanted with LNP/NVT

**Fig. 6:**
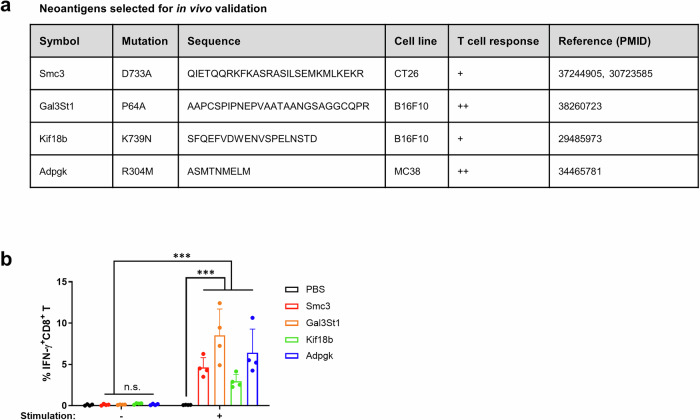
Neoantigen-specific T cell responses induced by NVT/LNP vaccine. **a** Neoantigen candidates selected for in vivo validation. **b** CD8^+^ T cell responses against various neoantigens induced by NVT/LNP vaccination. Each neoantigen peptide was administered to naive mice in a prime-boost schedule (1-week interval). PBMCs were collected 7 days after the final vaccination and restimulated with the corresponding peptide to evaluate neoantigen-specific CD8^+^ T cell responses. *n* = 4. The data are presented as mean ± SD. Statistical analyses were performed using one-way ANOVA with Tukey’s multiple comparisons test. ***P < 0.001; n.s. not significant.

One practical application of peptide vaccines is the development of neoantigen vaccines, a form of personalized cancer treatment. In this approach, epitopes from the mutated region are identified, synthesized into peptides, and combined with an appropriate adjuvant for administration. Recent clinical studies suggest that these neoantigen vaccines may serve as effective adjuvant therapies^31^. To investigate whether NVT/LNP can also act as an adjuvant for neoantigen vaccines, tests were conducted using several known mouse neoantigen epitopes (Fig. 6a). Each epitope was administered twice, with a one-week interval, using NVT/LNP as the adjuvant. PBMCs were isolated one week after the final vaccination, and epitope-specific CD8^+^ T cell responses were measured. The results showed a significant increase in epitope-specific CD8^+^ T cell responses for all neoantigen epitopes tested (Fig. 6b). This study demonstrates the potential of NVT/LNP as an adjuvant for neoantigen vaccines.

## Discussion

Although vaccines that induce protection through antibodies are routinely used in clinical practice, there remains a need for vaccines that elicit potent and sustained T cell immunity for specific infections, such as chronic diseases like malaria^32^, tuberculosis^33^, and chronic viral infections^34^, as well as for cancer treatment^35^. This study confirmed that encapsulating NVT in lipid nanoparticles induces a robust cellular immune response. When comparing the ability of NVT/LNP to induce cellular immunity with that of the adjuvant used in a previous clinical trial, we found that NVT/LNP exhibited significantly greater efficacy. Specifically, compared to the mRNA vaccine platform, which currently focuses on therapeutic vaccines targeting neoantigens^36–38^, NVT/LNP demonstrated superior performance, at least in terms of inducing target epitope-specific cellular immune responses. However, it is challenging to simultaneously compare the various advantages and disadvantages of each platform due to their differing modalities. Additionally, we experimentally demonstrated that a strong cellular immune response can be triggered against tumor-associated antigens (TAAs), which may exhibit self-tolerance, as well as neoantigen epitopes that are gaining attention for personalized treatment, confirming that this phenomenon is universal. Finally, when NVT/LNP was employed, a robust immune response was observed even without the conjugation process for epitopes. These findings are encouraging, considering the manufacturing and quality control disadvantages associated with the conjugated vaccine^39^.

We experimentally demonstrated that NVT/LNP increases intracellular uptake and activates TLR3 more effectively. In addition to the increased uptake, we hypothesized that enhanced resistance to nucleases would lead to greater stability and stronger efficacy. However, when comparing resistance to RNase A, we found that the stability was not significantly different from that of naked NVT (Supplementary Fig. 8). Therefore, the improved effect of NVT/LNP is attributed not to enhanced stability of NVT but to its efficient uptake by target cells. If an immunostimulant like NVT is not effectively decomposed in the body and remains for an extended period, it may enhance efficacy but could also pose risks of immune-related toxicity, such as cytokine syndrome^40^. In the case of NVT/LNP, such issues are not anticipated. Instead, it is expected to be more advantageous in terms of safety, as the drug’s potency increases through improved uptake efficiency, allowing for a smaller dosage.

In addition to NVT, several immunostimulants act within cells, including Poly(I:C), CpG, and STING. Studies have shown that their efficacy improves when encapsulated in LNPs^41–45^. This study shares similarities with previous research but also presents notable differences. First, we focused on cellular immune responses in this study. We believe that the primary role of immunostimulant adjuvants is to induce cellular immunity. Therefore, we concentrated on this aspect and applied it to the peptide vaccine platform that most requires it, ultimately finding that it effectively contributes to outcomes in anticancer and chronic virus treatments. Second, we confirmed variations in efficacy based on the ionized lipid used to formulate the LNPs. Specifically, we examined the immunogenicity associated with different types of ionized lipids in LNP composition. We prepared NVT/LNP using the cationic lipid DOTAP and the ionizable lipid SM-102, which is utilized in Moderna’s mRNA vaccine. Both lipids were well encapsulated and exhibited higher activity in vitro than naked NVT. However, the activity patterns differed in vivo. For DOTAP/LNP, unlike SM/LNP, DC activation in the spleen was reduced, as was the ability to induce systemic cellular immunity. To explore whether these differences would hold with other ionized lipids, we tested NVT with several commercially available options, including DODMA, DODAP, DORI, DLIN-KC2, and DLIN-MC3. We observed that DODMA, DODAP, and DORI exhibited behavior similar to DOTAP, while DLIN-KC2 and DLIN-MC3 resembled SM-102 (Supplementary Figs. 4–6). Several factors may explain these differences related to the ionized lipids. First, endosomal escape after intracellular uptake should be considered. However, based on our findings, where DOTAP/LNP demonstrated higher activity in in vitro reporter cell assays, endosomal escape does not appear to be the primary reason. Instead, the observed differences seem to stem from variations in the body distribution of LNPs formulated with each ionizable lipid. Our results also indicated that when administered intramuscularly to mice, NVT/SM-LNP elicited greater DC activation in the spleen compared to NVT/DOTAP-LNP. Furthermore, ex vivo imaging using fluorescent NVT confirmed that NVT/SM-LNP reached the spleen more effectively (Supplementary Fig. 9). Additionally, research indicating that mRNA expression distribution varies according to the charge differences of LNPs based on the ionized lipids supports this claim^46^. To our knowledge, this is the first study to confirm differences in immune-inducing ability based on the ionized lipid used for encapsulating an immunostimulant in LNPs.

In conclusion, encapsulation of NVT, a dsRNA-based TLR3 agonist, in lipid nanoparticles (LNPs) led to a robust induction of cellular immunity by enhancing uptake into target cells. Notably, the in vivo immune response varied depending on the ionizable lipid used, emphasizing the importance of lipid selection for optimal encapsulation. When formulated with suitable ionizable lipids and applied as an adjuvant for peptide vaccines, NVT/LNP demonstrated superior efficacy compared to existing adjuvants, resulting in strong therapeutic outcomes in multiple anticancer and chronic viral infection models. These findings provide evidence that NVT/LNP is a potent adjuvant platform for vaccines aimed at eliciting cellular immunity.

## Methods

### Reagents and mice

The ionizable lipids 1,2-dioleoyl-3-dimethylammonium propane (DODAP), 1,2-dioleyloxy-3-dimethylaminopropane (DODMA), (6Z,9Z,28Z,31Z)-heptatriaconta-6,9,28,31-tetraen-19-yl 4-(dimethylamino)butanoate (DLin-MC3-DMA), and 9-heptadecanyl 8-((2-hydroxyethyl)[6-oxo-6-(undecyloxy)hexyl]amino)octanoate (SM-102) were purchased from BroadPharm (San Diego, CA, USA). N,N-dimethyl-2,2-di-(9Z,12Z)-9,12-octadecadien-1-yl-1,3-dioxolane-4-ethanamine (DLin-KC2-DMA) was purchased from Cayman Chemical (Ann Arbor, MI, USA). The cationic lipid 1,2-dioleoyl-3-trimethylammonium-propane (DOTAP) was also purchased from BroadPharm (San Diego, CA, USA). Cholest-5-en-3β-ol (cholesterol) was obtained from Sigma-Aldrich (St. Louis, MO, USA). 1,2-Distearoyl-sn-glycero-3-phosphocholine (DSPC) and 1,2-dimyristoyl-rac-glycero-3-methoxypolyethylene glycol-2000 (DMG-PEG 2000) were purchased from Avanti Polar Lipids (Alabaster, AL, USA). 1,1’-Dioctadecyl-3,3,3’,3’-tetramethylindotricarbocyanine iodide (DiR) was obtained from Invitrogen™ (Thermo Fisher Scientific, Waltham, MA, USA). A fixable viability dye and PerCP-eF710 anti-mouse B220 antibody were purchased from Invitrogen (Thermo Fisher Scientific, Waltham, MA, USA). PE/Cyanine7 anti-mouse CD4, FITC anti-mouse CD8a, Alexa Fluor 700 anti-mouse CD8a, APC anti-mouse CD11c, APC anti-mouse CD44, PE anti-mouse CD80, PE/Cyanine5 anti-mouse CD86, PE/Cyanine7 anti-mouse MHC-II, and FITC anti-mouse XCR1 antibodies were purchased from BioLegend (San Diego, CA, USA). CpG ODN 2395 (5′-TCGTCGTTTTCGGCGCGCGCCG-3′) was obtained from Integrated DNA Technologies (IDT, Coralville, IA, USA). Poly(I:C)-HMW VacciGrade and Incomplete Freund’s Adjuvant (IFA) were purchased from InvivoGen (San Diego, CA, USA). GM-CSF was purchased from PeproTech (Rocky Hill, NJ, USA). All peptides were synthesized to a purity of 90% or higher by Dandicure (Ochang, Korea) and stored in lyophilized form. C57BL/6 and BALB/c female mice, aged 6 to 8 weeks, were purchased from Samtako Bio Korea (Kyounggi, Korea) and housed at the NA Vaccine Institute (NAVI) animal facility (Seoul, Korea). The mice were given a sterile, commercial diet and had access to water *ad libitum*. All procedures in this study were reviewed and approved by the Ethics Committee and Institutional Animal Care and Use Committee of NAVI (Permit Number: NAVI-2024-0005).

### Preparation of NVT and lipid nanoparticles (LNPs)

NVT was produced by in vitro transcription as previously described in ref. ^2^. For LNP formulation, the aqueous phase, containing RNA, and the organic phase, and lipids, were prepared separately before microfluidic mixing. Two types of RNA were used in the aqueous phase: double-stranded RNA NexaVant (NVT) and single-stranded OVA mRNA. The 5-methoxyuridine (5moU)-modified OVA mRNA was purchased from ApexBio Technology (Cat. No. R1028; Houston, TX, USA). The aqueous phase was prepared in 100 mM sodium acetate buffer (pH 5.0), with either NVT or 5moU-OVA mRNA dissolved to achieve nitrogen-to-phosphate (N/P) ratios of 1:1, 2:1, 4:1, and 6:1. For the organic phase, lipid stock solutions were prepared in ethanol and stored at concentrations of 10 or 50 mg/ml. The lipid mixture consisted of 46.3% ionizable or cationic lipid, 9.4% DSPC, 42.7% cholesterol, and 1.6% DMG-PEG 2000, resulting in a final lipid concentration of 18 mM. The aqueous and organic phases were mixed using the Mixer-3 or Mixer-4 chip on the NanoGenerator Flex-M Nanoparticle Synthesis System (Precigenome, San Jose, CA) at an aqueous-to-organic flow rate ratio of 3:1. Total flow rates were set at 5 mL/min for the Mixer-4 chip and 12 mL/min for the Mixer-3 chip. DiR-labeled LNPs were prepared by incorporating DiR dye (1 mol% of total lipids) into the organic phase before mixing. Immediately following nanoparticle synthesis, LNPs were dialyzed against an excess volume of phosphate-buffered saline (PBS, pH 7.4) using Slide-A-Lyzer™ dialysis cassettes with a 20 kDa molecular weight cutoff for 16–24 hours. After dialysis, the LNPs were filtered through 0.22 μm polyethersulfone (PES) filters, concentrated to the desired volume using Amicon Ultra-15 centrifugal filters (30 kDa cutoff), and stored at 4°C until use.

### LNP characterization

Particle size and polydispersity index (PDI) of LNP formulations were measured using a Zetasizer Pro (Malvern Panalytical Ltd., Malvern, UK) at 25 °C. The instrument was equipped with a 633 nm He–Ne laser and utilized a 173° backscatter detection angle. Before measurement, LNP samples were diluted 1:100 (v/v) in phosphate-buffered saline (PBS). RNA encapsulation efficiency of the LNPs was assessed using the RiboGreen™ mRNA quantification assay kit, following the manufacturer’s instructions. Fluorescence intensity of the RiboGreen dye bound to RNA was measured in the absence and presence of 0.1% Triton X-100, a membrane permeabilization agent, to distinguish between encapsulated and free RNA. Fluorescence was recorded using a microplate reader with excitation and emission wavelengths set at 480 nm and 520 nm, respectively.

### RNA extraction from RNA-LNPs

RNA was extracted from the RNA-LNP formulation by isopropanol precipitation. Specifically, 100 µL of mRNA-LNP was diluted 10-fold in 900 µL of isopropanol containing 60 mM ammonium acetate, briefly vortexed, and centrifuged at 14,000 g for 15 min at 4 °C. The supernatant was discarded, and the pellet was washed with 1 mL of isopropanol, vortexed, and centrifuged again at 4 °C. The resulting pellet was dried in vacuo and resuspended in 100 µL of RNase-free water at room temperature.

### Agarose gel electrophoresis

NVT and NVT/LNP samples were loaded onto a 1% agarose gel prepared with 1× TAE buffer containing RedSafe Nucleic Acid Staining Solution (iNtRON Biotechnology, Seongnam, Korea). Electrophoresis was performed at 100 V for 30 min, and the bands were visualized using a Gel Imaging System (CG-500, Davinch Biotech, Korea). For electrophoresis of NVT/LNP, NVT was extracted from the NVT/LNP formulation using ammonium acetate and isopropanol. The purified NVT was subsequently loaded onto the gel.

### TLR3 activation assay with a reporter cell line

Human TLR3-expressing HEK 293 cells (Invivogen, San Diego, CA, USA) were cultured in Dulbecco’s modified Eagle medium supplemented with 4.5 g/l glucose, 2 mM L-glutamine, 10% heat-inactivated fetal bovine serum (FBS), and 1% penicillin/streptomycin in the presence of blasticidin (Sigma-Aldrich, St. Louis, MO, USA) (30 μg/ml), zeocin (Invivogen, San Diego, CA, USA) (100 μg/ml), and normocin (Invivogen) (100 μg/ml). To assess TLR3 activation by NVT and NVT/LNP, 100 µl of 5 × 10^4^ cells were mixed with 100 µl of various concentrations of NVT or NVT/LNP in HEK-Blue™ detection medium in 96-well culture plates. The mixtures were incubated for 16 h at 37 °C with 5% CO_2_, and the absorbance was measured at 655 nm.

### Cell uptake assay

RAW264.7 cells (Korean Cell Line Bank(KCLB), Seoul, Korea) were seeded at a density of 2.5 × 10⁵ cells per well in 24-well plates and incubated overnight. The following day, IR750-labeled NVT, NVT/DOTAP-LNP, or NVT/SM-LNP were added at a concentration of 1 μg/ml and incubated for 1 or 4 h. After incubation, cells were washed twice with PBS and harvested. The mean fluorescence intensity (MFI) of IR750 was measured using NovoCyte flow cytometry (ACEA Biosciences, San Diego, CA, USA).

### Innate immune response

C57BL/6 mice were injected intramuscularly in the thigh muscle of the right hind limb with various doses of NVT, NVT/LNP, or empty LNP. Serum samples were collected 6 and 24 days after injection, and the concentrations of IFN-β and IL-6 in the serum were measured using ELISA according to the manufacturer’s instructions. To analyze DC activation, the draining inguinal lymph node (iLN) from the right hind leg and the spleen were collected 24 h post-injection and dissociated into single-cell suspensions. The cells were stained with a fixable viability dye to distinguish live from dead cells, followed by surface staining with the following antibodies: APC anti-mouse CD11c, PE/Cyanine7 anti-mouse MHC-II, FITC anti-mouse XCR1, PerCP-eF710 anti-mouse B220, AF700 anti-mouse CD8, PE anti-mouse CD80, and PE/Cyanine5 anti-mouse CD86. Cells were gated as cDC1 (CD11c^+^MHC-II^+^XCR1^+^B220^-^), cDC2 (CD11c^+^MHC-II^+^XCR1, and pDC (CD11c^+^MHC-II^+^XCR1^-^B220^+^) (Supplementary Fig. 10), and the intensity of surface expression for CD80 and CD86 was measured using flow cytometry.

### Peptide-specific T cell response

C57BL/6 mice were vaccinated intramuscularly in the thigh muscle of the right hind limb three times at one-week intervals with HPV16 E7_49-57_ (RAHYNIVTF, E7), OVA CD8 pep_257-264_ (SIINFEKL, OVA), Smc3_720-746_ (QIETQQRKFKASRASILSEMKMLKEKR, Smc3), Gal3St1_49-75_ (AAPCSPIPNEPVAATAANGSAGGCQPR, Gal3St1), Kif18b_730-748_ (SFQEFVDWENVSPELNSTD, Kif18b), Yju2_4-27_ (RKVLNKYYPPDSDPSKIPKLKLPK, Yju2), Adpgk_299-307_ (ASMTNMELM, Adpgk), lymphocytic choriomeningitis virus (LCMV) GP_33-41_ (KAVYNFATC, GP33), or GP_66-71_ (DIYKGVYQFKSV, GP66) peptide antigens formulated with each adjuvant. One week after the final vaccination, PBMCs were isolated from cheek blood collected from mice and restimulated overnight with 20 μg/ml of each peptide. Then, GolgiPlug (BD Biosciences, Franklin Lakes, NJ, USA) was added to the cells at 1 μg/ml and incubated for an additional 4–6 h to block intracellular cytokine secretion. The cells were stained with a viability dye, followed by surface staining with PE/Cyanine7 anti-mouse CD4, FITC anti-mouse CD8, and APC anti-mouse CD44 antibodies for 20 min at 4 °C. For intracellular staining, the cells were fixed and permeabilized using Cytofix/Cytoperm solution (BD Biosciences) and then stained with PE anti-mouse IFN-γ antibodies for 20 min at 4 °C. Finally, the cells were washed, resuspended in FACS buffer (PBS containing 1% FBS and 0.1% NaN), and CD4⁺CD44⁺IFN-γ⁺ and CD8⁺CD44⁺IFN-γ⁺ T cells were analyzed by flow cytometry to evaluate antigen-specific T cell responses (Supplementary Fig. 11).

### Tumor models

C57BL/6 mice were subcutaneously injected with 5 × 10^5^ TC-1-Luc or B16F10-OVA cells to establish the subcutaneous tumor model. Tumor-bearing mice were randomly assigned to different experimental groups and vaccinated intramuscularly with the indicated vaccines on days 7, 14, and 21 after tumor implantation. Subcutaneous tumor size was measured regularly using calipers and in vivo imaging system, and tumor volume was calculated using the formula: Volume = (length × width × height × 3.14)/6. To induce lung metastasis, C57BL/6 mice were intravenously injected with 5 × 10^5^ TC-1-Luc or B16F10-OVA cells. Tumor metastasis was assessed either by quantifying luminescence intensity to estimate tumor burden or by visual inspection of lung tissues following sacrifice at a predefined time point. Lung tumor nodules were counted by size, and the metastatic burden was evaluated. C57BL/6 mice were intravaginally inoculated with 5 × 10⁵ TC-1-Luc cells to induce cervical cancer. Mice were vaccinated with E7 peptide-formulated NVT/LNP or control vaccines intramuscularly on days 7, 14, and 21. Cervical tumor progression was monitored using in vivo luminescence imaging on day 25. Tumor burden was quantified by measuring luminescence signal intensity at multiple time points. Peripheral blood mononuclear cells (PBMCs) were isolated, and Ag-specific CD8^+^ T cell responses were measured using flow cytometry.

### In vivo bioluminescence imaging

In vivo bioluminescence imaging was conducted using the VISQUE® Smart-LF imaging system (Chayon Laboratories, Seoul, Korea). Mice bearing luciferase-expressing tumors were anesthetized with isoflurane and administered D-luciferin potassium salt (40 mg/kg body weight; PerkinElmer, Waltham, MA, USA) via intraperitoneal injection. Bioluminescent signals were acquired approximately 10 min post-injection. Quantification of signal intensity was performed using the manufacturer’s VISQUE Imaging Software, and total photon flux (photons/sec) was calculated from defined regions of interest (ROIs).

### Gross morphological imaging

Gross morphology of mice and lungs was imaged using a Sony ILCE-5100 digital camera (Sony, Tokyo, Japan) under standardized lighting conditions. Captured images were adjusted for brightness ( + 20%) using Microsoft PowerPoint (Microsoft Corporation, Redmond, WA, USA).

### Depletion of CD4^+^ and CD8^+^ T cells

For T cell depletion, *InVivo*MAb anti-CD4 monoclonal antibody (clone GK1.5, BE0003-1, BioXCell, Lebanon, NH, USA) and *InVivo*MAb anti-CD8α monoclonal antibody (clone 2.43, BE0061, BioXCell) were administered intraperitoneally. An isotype control antibody (*InVivo*MAb rat IgG2b, clone LTF-2, BE0090, BioXCell) was used as a negative control. Mice received 200 µg of antibody per dose, twice weekly for a total of six doses. The efficiency of T cell depletion was confirmed by flow cytometric analysis of CD4^+^ and CD8^+^ T cell levels in PBMCs (data not shown).

### Therapeutic LCMV models

The Clone 13 (Cl13) strain of lymphocytic choriomeningitis virus (LCMV) was propagated in baby hamster kidney (BHK) cells by Prof. Young-Jin Seo (Chungang University, Seoul, Korea). C57BL/6 mice were intravenously infected with 2 × 10^6^ plaque-forming units (PFU) of LCMV Cl13. Fourteen days after infection, mice were intramuscularly immunized with NVT/LNP-formulated GP33 peptide at weekly intervals for a total of three doses. One week after the final vaccination, viral titers in the blood were quantified using a plaque assay with Vero (African green monkey kidney) cells.

### Peptide-CRM197 conjugation

TRP2 peptides were conjugated to the carrier protein CRM197 (a nontoxic mutant of diphtheria toxin; Eubiologics, Chuncheon-si, Korea) using a sulfhydryl–maleimide coupling strategy. Synthetic peptides were designed with an additional C-terminal cysteine to allow site-specific conjugation. CRM197 was incubated with a 20-fold molar excess of sulfo-SMCC (sulfosuccinimidyl 4-(N-maleimidomethyl)cyclohexane-1-carboxylate; Thermo Fisher Scientific, Waltham, MA, USA) in PBS containing 1 mM EDTA (pH 7.4) for 1 h at room temperature with gentle agitation to introduce maleimide groups. Unreacted crosslinker was removed using Zeba Spin Desalting Columns (Thermo Fisher Scientific). The cysteine-modified peptide was then reacted with maleimide-activated CRM197 at a 20:1 molar ratio (peptide:CRM197) in PBS with 1 mM EDTA and incubated for 1 h at room temperature under gentle agitation. TRP2–CRM197 conjugates were purified by ultrafiltration (Amicon Ultra, 10 kDa cutoff; Millipore, Burlington, MA, USA) to remove free peptides and stored at −80 °C until use. Conjugation efficiency and purity were assessed by 10% SDS-PAGE.

### Statistical analysis

Statistical analyses were conducted using GraphPad Prism version 10.4.0 for Windows (GraphPad Software, La Jolla, CA, USA; www.graphpad.com). A one-way analysis of variance (ANOVA) followed by Tukey’s multiple comparison test was used to determine significant differences among multiple groups. Survival data were analyzed using Kaplan–Meier survival analysis. A *p*-value of less than 0.05 was considered statistically significant.

### Ethics statement

All animal procedures were performed following the protocols set by the Korean Food and Drug Administration. To reduce stress and ensure proper sedation during experimental procedures and euthanasia, animals were anesthetized using inhaled isoflurane. For euthanasia, deep anesthesia was achieved with 5% isoflurane, and the depth of anesthesia was verified by the total absence of reflex responses. The experimental protocols received approval from the Institutional Animal Care and Use Committee (IACUC) of the NA Vaccine Institute (NAVI, Seoul, Korea), under approval number 2024-0007.

## Supplementary information

Supplementary Information

## Data Availability

All data supporting the findings of this study are available within the article and its supplementary information files. No datasets requiring deposition were generated, as all peptides were derived from known protein sequences.
