# Supplementary material for: Lipid nanoparticle encapsulated TLR3 agonist adjuvant elicits potent T cell immunity against cancer and viruses
Source: NPJ Vaccines. 2025 Dec 23;11:26. doi: 10.1038/s41541-025-01349-w (PMC12848299; doi:10.1038/s41541-025-01349-w)
Supplement: Supplementary file 1 — Supplementary Information [file 41541_2025_1349_MOESM1_ESM.pdf]

Supplementary Information

Lipid nanoparticle encapsulated TLR3 agonist adjuvant elicits potent T cell immunity against cancer and viruses

Kwang Hyun Ko[#], Seung-Hwan Lee[#], Young-Ho Choi, Soon Myung Kang, Hyun-Suk Yang, So Min Lee, Eun Bi Jo, Hyun Shik Bae, Seung-Beom Hong, Dong-Ho Kim*, and Seung Bin Cha*

[1]R&D Center, NA Vaccine Institute, Seoul 05854, Republic of Korea

[#] These authors are contributed equally

[*] These authors are contributed equally

Supplementary Figures

Supplementary Fig. 1. Original, uncropped agarose gel images corresponding to Fig. 1b.

Supplementary Fig. 2. Flow cytometry pseudocolor plots and histograms of CD80 and CD86 in splenic cDC1

Supplementary Fig. 3. DC activation according to dose of NVT/LNP

Supplementary Fig. 4. Characterization of NVT/LNPs prepared with various ionized lipids

Supplementary Fig. 5. Innate immune response of NVT/LNPs prepared with various ionized lipids

Supplementary Fig. 6. Adaptive immune responses of NVT/LNPs prepared with various ionized lipids

Supplementary Fig. 7. Validation of $CD4^+$ and $CD8^+$ T cell depletion

Supplementary Fig. 8. Stability of NVT and NVT/LNP against RNase A

Supplementary Fig. 9. Differences in LNP distribution to the spleen depending on the ionizable lipids formulated in NVT/LNP

Supplementary Fig. 10. Gating strategy for DC subset analysis

Supplementary Fig. 11. Gating strategy for T cell response analysis

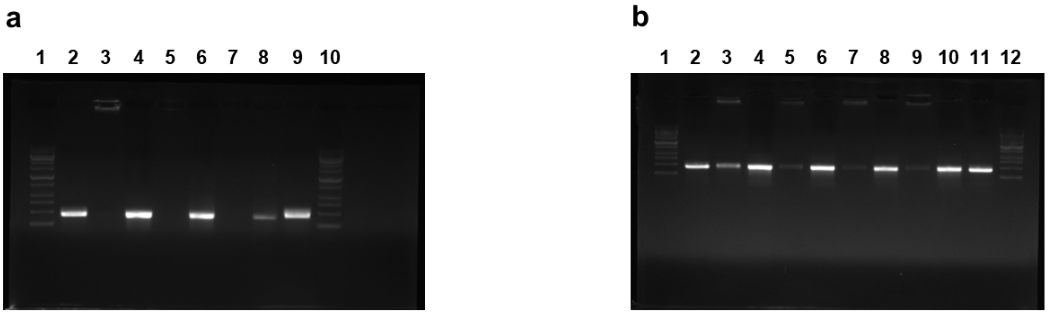

Lane #1: Marker
Lane #2: NVT
Lane #3: NVT/DOTAP-LNP N/P 2 batch#1
Lane #4: NVT/DOTAP-LNP N/P 2 batch#1 (NVT extraction)
Lane #5: NVT/DOTAP-LNP N/P 2 batch#2
Lane #6: NVT/DOTAP-LNP N/P 2 batch#2 (NVT extraction)
Lane #7: Empty
Lane #8: NVT/DOTAP-LNP N/P 4
Lane #9: NVT/DOTAP-LNP N/P 4 (NVT extraction)
Lane #10: Marker

Lane #1: Marker
Lane #2: NVT
Lane #3: NVT/SM-LNP N/P 1
Lane #4: NVT/SM-LNP N/P 1 (NVT extraction)
Lane #5: NVT/SM-LNP N/P 2
Lane #6: NVT/SM-LNP N/P 2 (NVT extraction)
Lane #7: NVT/SM-LNP N/P 4
Lane #8: NVT/SM-LNP N/P 4 (NVT extraction)
Lane #9: NVT/SM-LNP N/P 6
Lane #10: NVT/SM-LNP N/P 6 (NVT extraction)
Lane #11: NVT
Lane #12: Marker

Supplementary Fig. 1. Original, uncropped agarose gel images corresponding to Fig. 1b. **a** Agarose gel electrophoresis of NVT/DOTAP-LNP at two N/P ratios, 2 and 4. Two separate batches were tested for N/P 2. Lanes are labeled as follows: Lane #1 and #10, molecular weight markers; Lane #2 and #11, free NVT; Lane #3 and #5, NVT/DOTAP-LNP N/P 2 batch #1 and batch #2; Lane #4 and #6, corresponding NVT extraction samples from each batch; Lane #7, empty control; Lane #8, NVT/DOTAP-LNP N/P 4; Lane #9, corresponding NVT extraction from N/P 4. **b** Agarose gel electrophoresis of NVT/SM-LNP at various N/P ratios. Lanes include: Lane #1 and #12, molecular weight markers; Lane #2 and #11, free NVT; Lanes #3, #5, #7, and #9, NVT/SM-LNP at N/P ratios 1, 2, 4, and 6; Lanes #4, #6, #8, and #10, corresponding NVT extraction samples. NVT extraction was performed using ammonium acetate and isopropanol. All lanes and bands shown in Figure 1b are included here with no cropping or contrast adjustment. The main figure shows a subset of these lanes for clarity.

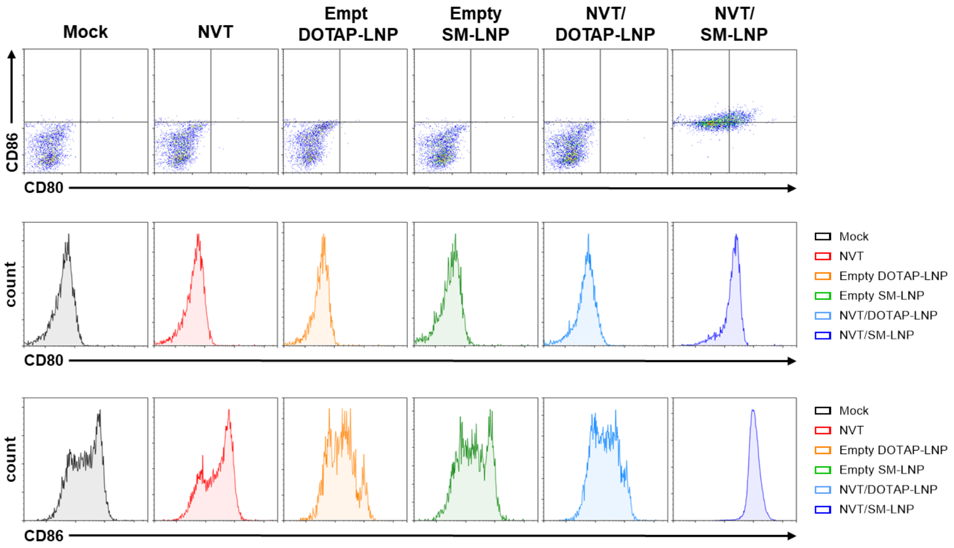

Supplementary Fig. 2. Flow cytometry pseudocolor plots and histograms of CD80 and CD86 in splenic cDC1. C57BL/6 mice were immunized intramuscularly with 10 μg of NVT, NVT/DOTAP-LNP, NVT/SM-LNP, or equivalent amounts of empty DOTAP-LNP or empty SM-LNP. Flow cytometry pseudocolor dot plots and histograms show CD80 and CD86 expression in splenic cDC1 cells 24 hours post-immunization. The top row depicts pseudocolor plots of CD80 versus CD86, and the middle and bottom rows show histograms of CD80 and CD86 expression, respectively, for each treatment group.

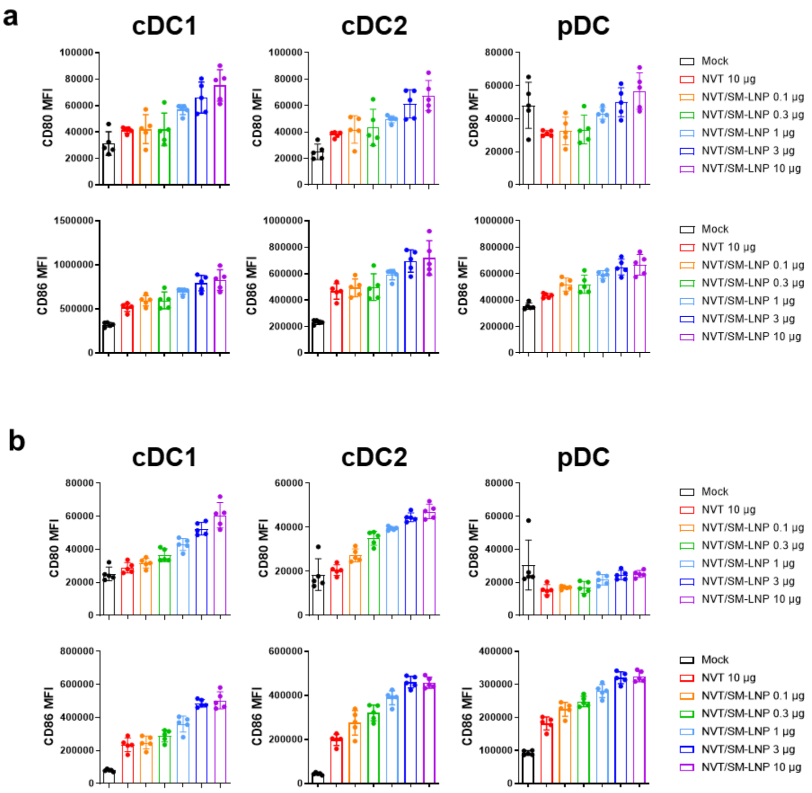

Supplementary Fig. 3. DC activation according to dose of NVT/LNP. a, b Mice were immunized intramuscularly with either 10 μg of NVT or various doses of NVT/SM-LNP. The MFI of CD80 and CD86 in the cDC1 (CD11c^{+}MHC-II^{+}XCR1^{+}CD8^{+}), cDC2 (CD11c^{+}MHC-II^{+}XCR1^{-}), and pDC (CD11c^{+}MHC-II^{+}B220^{+}) subpopulations in (**a**) inguinal lymph nodes and (**b**) spleen at 24 hours post-immunization was measured by flow cytometry. n = 5.

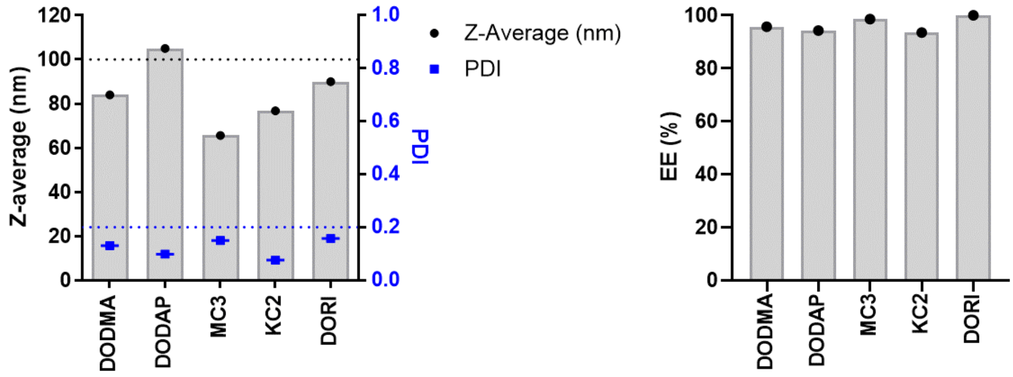

Supplementary Fig. 4. Characterization of NVT/LNPs prepared with various ionized lipids. Particle size, polydispersity index (PDI), and encapsulation efficiency (EE) of NVT/DODMA-LNP, NVT/DODAP-LNP, NVT/MC3-LNP, NVT/KC2-LNP, NVT/DORI-LNP were characterized by dynamic light scattering.

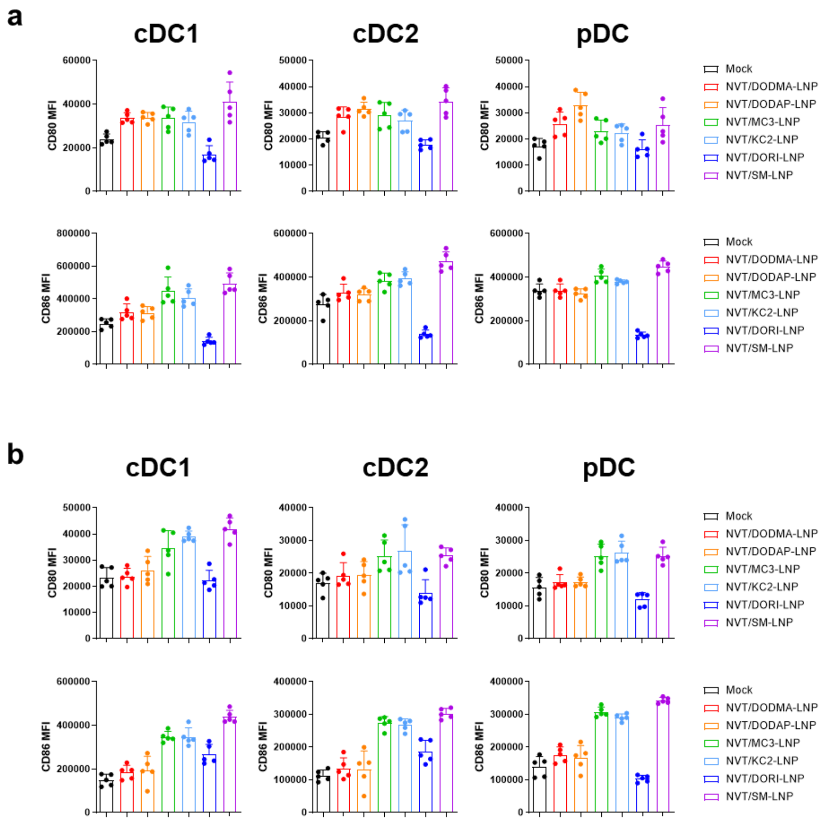

Supplementary Fig. 5. Innate immune response of NVT/LNPs prepared with various ionized lipids. a, b Mice were immunized intramuscularly with 10 μg of NVT/DODMA-LNP, NVT/DODAP-LNP, NVT/MC3-LNP, NVT/KC2-LNP, NVT/DORI-LNP, and NVT/SM-LNP. The MFI of CD80 and CD86 in the cDC1 (CD11c$^+$, MHC-II$^+$, XCR1$^+$, CD8$^+$), cDC2 (CD11c$^+$, MHC-II$^+$, XCR1$^-$), and pDC (CD11c$^+$, MHC-II$^+$, B220$^+$) subpopulations in (a) inguinal lymph nodes and (b) spleen at 24 hours post-immunization was measured by flow cytometry (n = 5).

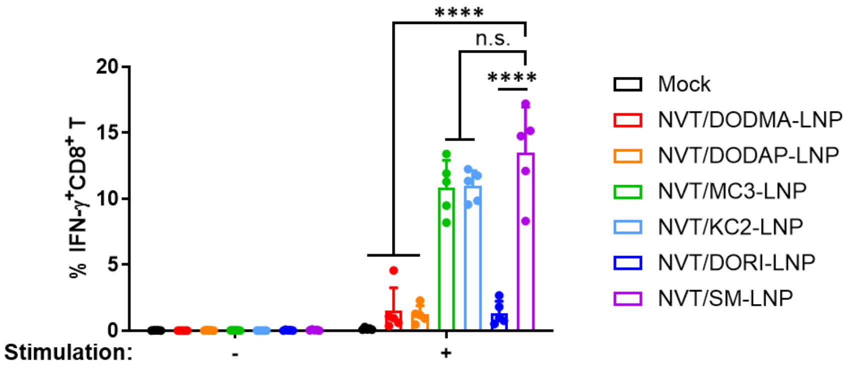

Supplementary Fig. 6. Adaptive immune responses of NVT/LNPs prepared with various ionized lipids. C57BL/6 mice were immunized intramuscularly with 10 µg of GP33 formulated with 10 µg of NVT/DODMA-LNP, NVT/DODAP-LNP, NVT/MC3-LNP, NVT/KC2-LNP, NVT/DORI-LNP, or NVT/SM-LNP on days 7, 14, and 21. One week after the final vaccination, GP33-specific T cell responses in PBMCs were measured using flow cytometry. The data are presented as mean ± SD. Statistical analyses were performed using one-way ANOVA with Tukey's multiple comparisons test. ****$P < 0.0001$; n.s., not significant.

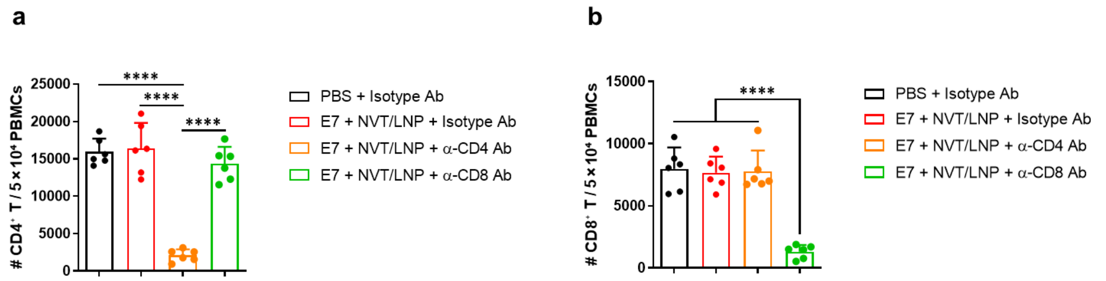

Supplementary Fig. 7. Validation of CD4$^+$ and CD8$^+$ T cell depletion. Tumor-bearing mice were vaccinated with NVT/LNP on days 7, 14, and 21, and anti-CD4 or anti-CD8 antibodies were administered intraperitoneally twice weekly starting on day 7 for six doses. PBMCs were collected on day 18 and analyzed by flow cytometry. Graphs show the numbers of (**a**) CD4$^+$ and (**b**) CD8$^+$ T cells, confirming effective depletion of the targeted populations.

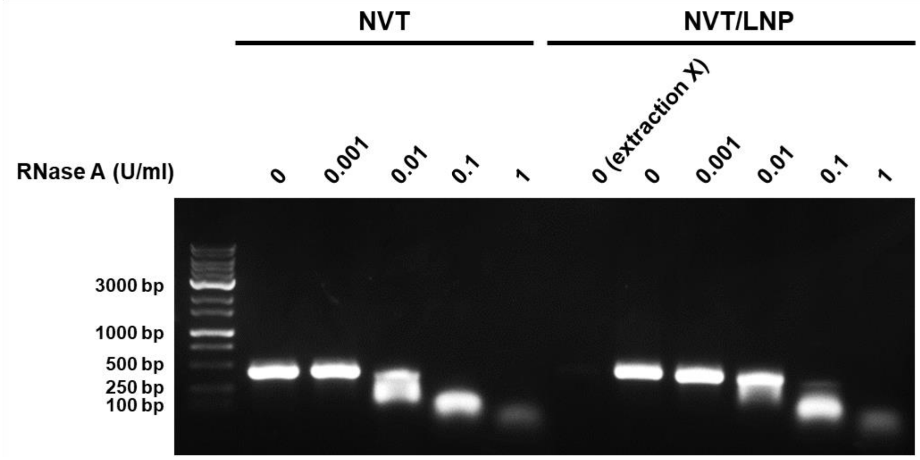

Supplementary Fig. 8. Stability of NVT and NVT/LNP against RNase A. NVT and NVT/LNP were incubated with 0.001, 0.01, 0.1, or 1 U/ml of RNase A at 37°C for 10 minutes, after which electrophoresis was performed on a 1% agarose gel. The NVT in the NVT/LNP was extracted using ammonium acetate and isopropanol.

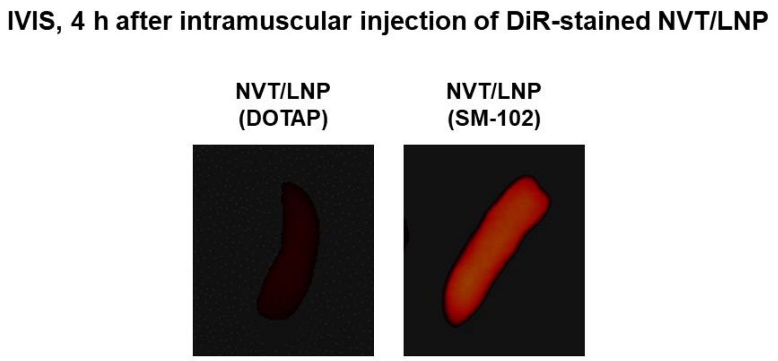

Supplementary Fig. 9. Differences in LNP distribution to the spleen depending on the ionizable lipids formulated in NVT/LNP. Naive mice were intramuscularly injected with DiR-labeled NVT/DOTAP-LNP or NVT/SM-LNP. Four hours after administration, spleens were collected and fluorescence signals were analyzed *ex vivo*. Representative data from three independent experiments are presented.

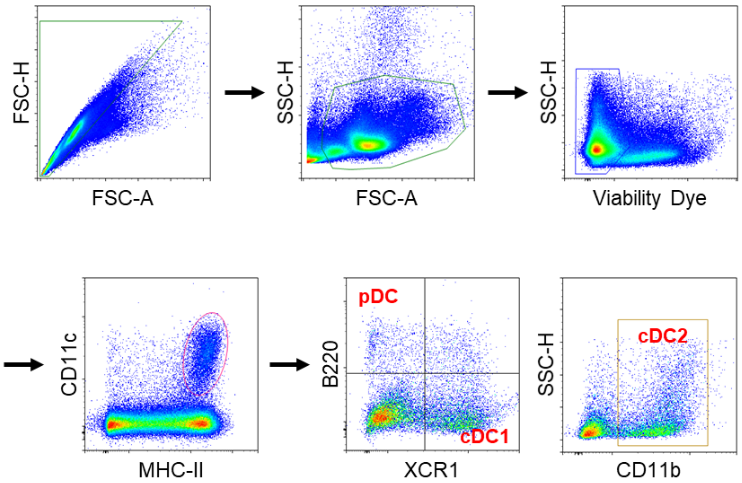

Supplementary Fig. 10. Gating strategy for DC subset analysis. Single cells were initially selected by gating on forward scatter area versus height (FSC-A vs. FSC-H). Debris was removed by excluding events with low forward and side scatter height (FSC-H and SSC-H) values. Viable cells were then identified by excluding dead cells stained with a fixable viability dye. Within the live cell population, $CD11c^{+}MHC\text{-}II^{+}$ cells, representing DCs, were first gated, and within this gated population, subsets were defined as follows: cDC1 as $XCR1^{+}B220^{-}$ cells, pDC as $XCR1^{-}B220^{+}$ cells, and cDC2 as $CD11b^{+}$ cells.

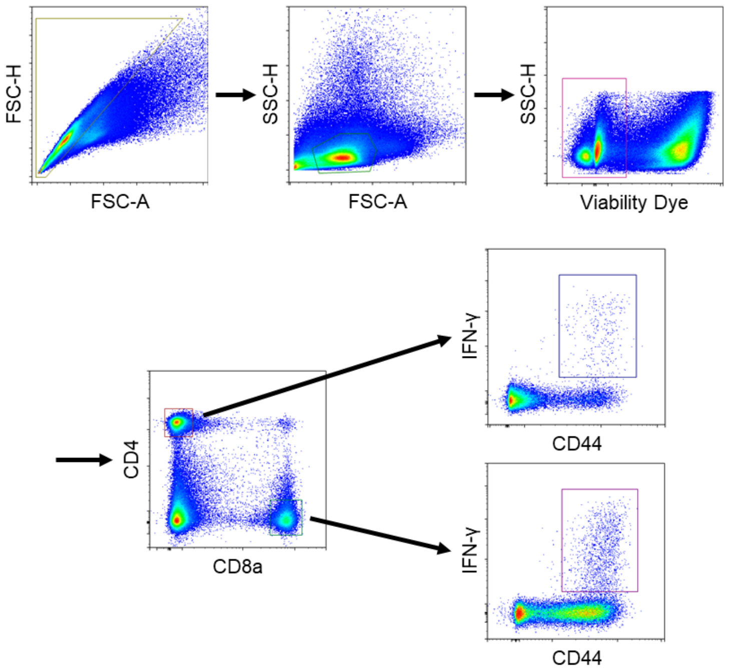

Supplementary Fig. 11. Gating strategy for T cell response analysis. To evaluate T cell responses, singlet cells were first selected using forward scatter area versus height (FSC-A vs. FSC-H). Lymphocytes were then gated based on their size and granularity (FSC and SSC). Live cells were identified by staining with a viability dye. $CD4^{+}CD8^{-}$ cells were designated as $CD4^{+}$ T cells, and $CD4^{-}CD8^{+}$ cells as $CD8^{+}$ T cells. T cell responses were assessed by analyzing $CD44^{+}IFN\text{-}\gamma^{+}$ cells within each T cell subset.
